# The influence of 3x3 bonded retainer on anterior crowding relapse in mandibular incisor extraction cases

**DOI:** 10.1590/2177-6709.26.6.e212081.oar

**Published:** 2021-12-15

**Authors:** Marcelo BERBERT, Paula COTRIN, Renata Cristina Gobbi de OLIVEIRA, Ricardo Gobbi de OLIVEIRA, Fabricio Pinelli VALARELLI, Marcos Roberto de FREITAS, Karina Maria Salvatore FREITAS

**Affiliations:** 1 Centro Universitário Ingá, Faculdade de Odontologia (Maringá/PR, Brazil); 2 Universidade de São Paulo, Faculdade de Odontologia de Bauru, Departamento de Odontologia (Bauru/SP, Brazil)

**Keywords:** Relapse, Stability, Tooth extraction

## Abstract

**Objective:**

To evaluate the 3x3 bonded retainer influence on the mandibular anterior crowding in cases treated with mandibular incisor extraction

**Methods:**

The sample comprised pretreatment, posttreatment and follow-up orthodontic records of 16 subjects (10 females and 6 males) with Class I malocclusion treated with extraction of a single mandibular incisor. The mean ages (± SD) at pretreatment, posttreatment and follow-up evaluation were 23.45 ± 9.14 years, 25.50 ± 8.95 years and 30.11 ± 8.59 years, respectively. The mean (± SD) treatment time and posttreatment evaluation time were 2.05 ± 0.45 years and 4.60 ± 1.85 years , respectively. Little irregularity index and interdental widths were evaluated using dental casts. The sample was divided into two subgroups, according to the presence of the 3x3 bonded retainer at follow-up.

**Results:**

The subgroup without 3x3 bonded retainer presented a greater relapse at the follow-up, when compared to 3x3 bonded retainer subgroup.

**Conclusion:**

There was a significant relapse in cases treated with mandibular incisor extraction at follow-up. The subgroup without 3x3 bonded retainer showed a significant relapse at the follow-up when compared to the retainer group.

## RESUMO

**Objetivo:**

Avaliar a influência da contenção fixa 3x3 no apinhamento anterior em casos tratados com extração de incisivo inferior.

**Métodos:**

A amostra foi constituída por arquivos ortodônticos das fases pré-tratamento, pós-tratamento e avaliação em longo prazo, de 16 pacientes (10 mulheres e 6 homens) portadores de má oclusão de Classe I tratados com a extração de um único incisivo inferior. As idades médias (± DP) pré-tratamento, pós-tratamento e na avaliação em longo prazo foram 23,45 ± 9,14 anos, 25,50 ± 8,95 anos e 30,11 ± 8,59 anos, respectivamente. O tempo médio (± DP) de tratamento e de acompanhamento em longo prazo foi 2,05 ± 0,45 anos e 4,60 ± 1,85 anos, respectivamente. Nos modelos de gesso, foram avaliados o Índice de Irregularidade de Little e as distâncias interdentárias. A amostra foi dividida em dois subgrupos, de acordo com a presença da contenção fixa 3x3 na avaliação em longo prazo.

**Resultados:**

O grupo sem contenção fixa apresentou uma maior recidiva na avaliação em longo prazo, quando comparado com o grupo que tinha a contenção fixa.

**Conclusão:**

Houve uma recidiva significativa nos casos tratados com extração de incisivo, na avaliação em longo prazo. O subgrupo sem contenção fixa 3x3 mostrou uma recidiva significativa em longo prazo, quando comparado ao grupo com contenção.

## INTRODUCTION

Retention and stability are always a concern for orthodontists. Maintaining a stable orthodontic treatment over the years posttreatment is a challenge. Several long-term retention studies evaluating the stability of different treatment modalities have reported that some relapse can be expected irrespective of initial malocclusion or type of treatment.^1^ ^-^ ^3^ Most of the researches is centered on the mandibular anterior crowding relapse.^4^ ^-^ ^6^ Long-term follow-up studies show that long-term response to mandibular anterior alignment is unpredictable; furthermore, parameters such as initial crowding, age, sex, Angle classification, maxillary and mandibular incisor proclination, horizontal and vertical growth amounts have not been useful in establishing a prognosis.^7^ ^,^ ^8^ It has also been shown that two thirds of the patients presented unsatisfactory mandibular anterior alignment after retention, and crowding continues to increase during the 10 to 20 years posttreatment.^7^ ^,^ ^9^

Mandibular anterior crowding is the most common malocclusion feature found in the population^10^ and several treatment modalities can be employed for treatment, such as distal movement of posterior teeth, lateral movement of canines, labial movement of incisors, interproximal enamel reduction, premolars extraction, incisors extractions or even a combination of the above mentioned. In cases treated nonextraction, crowding resolution is performed by an increase in arch perimeter, achieved by generalized expansion of the buccal segments, along with advancement of the mandibular incisors.^11^ Despite these changes may be consistent with certain treatment objectives; in others, they may be undesirable.

Mandibular incisor extraction is indicated in carefully selected cases to resolve crowding, especially when space requirements and facial esthetics do not call for greater dental movements. Incisor extraction is effective in treating Class I malocclusion in permanent dentition with moderate anterior mandibular crowding.^12^ ^-^ ^14^ The intentional extraction of a mandibular incisor can enable the orthodontist to produce enhanced functional occlusal and cosmetic results with minimal orthodontic manipulation.^15^ There are four classical indications for mandibular incisor extraction: anomalies in the number of anterior teeth; tooth size anomalies, ectopic eruption of incisors and moderate Class III malocclusions.^16^ Additionally, Brandt and Safirstein^17^ stated as advantage of incisor extraction the possibility of maintenance of intercanine width. It is assumed that keeping the general arch form increases the outcome stability, besides reducing the retention period.

It is known that occlusal relapse can be expected after active orthodontic treatment irrespective of long-term use of fixed retainers,^18^ while some authors^9^ ^,^ ^19^ ^,^ ^20^ state that a fixed retainer should be in place to ensure long-term mandibular anterior alignment. Few researches have been conducted to evaluate protocols and trends in orthodontic retention, and the quality of the available evidence is low. Regarding mandibular anterior teeth, there is a lack of published evidence to guide the clinical practice of orthodontic retention and relapse management.^21^ ^-^ ^24^ The aim of this study was to evaluate if the 3x3 bonded mandibular retainer influences the relapse of anterior crowding in cases treated with mandibular incisor extraction.

## MATERIAL AND METHODS

### MATERIAL

This retrospective study was approved by the Ethics in Human Research Committee at Centro Universitário Ingá under number 61629516.7.0000.5220.

Sample size calculation was performed based on an alpha significance level of 5% and beta of 20% to detect a minimum difference of 0.35mm with a standard deviation of 0.34mm for the Little Irregularity Index.^25^ Thus, the sample size calculation resulted in the need of 16 patients.

Data were collected according to the following inclusion criteria: Class I malocclusion patients with straight profile, mild to moderate mandibular anterior crowding, maxillary teeth generally well aligned, with the dental midline coincident with the facial midline, complete permanent dentition up to first permanent molars at the beginning of treatment, no dental agenesis, no tooth shape or number abnormalities, and no previous orthodontic treatment performed with mandibular incisor extraction.

The total sample comprised pre- and posttreatment orthodontic records (dental casts and intraoral photographs) from 16 patients (10 females, 6 males). The mean (±SD) pretreatment (T_1_), posttreatment (T_2_) and last follow-up (T_3_) ages were 23.45 ± 9.14, 25.50± 8.95 and 30.11 ± 8.59 years, respectively. The mean treatment time was 2.05 ± 0.45 years, and the mean follow-up time was 4.6 ± 1.85 years (Table 1).

**Table 1:** Comparison of the Little Irregularity Index, overjet, overbite and interdental widths in the three stages (n = 16, repeated measures ANOVA and Tukey tests).

Variables (mm)	Initial (T_1_)	Final (T_2_)	Follow-up (T_3_)	*p*
	Mean (SD)	Mean (SD)	Mean (SD)	
Little	8.89 (1.29)^A^	0.25 (0.12)^B^	1.67 (1.03)^C^	0.000*
Overjet	3.96 (0.94)^A^	2.96 (0.46)^B^	3.18 (0.65)^B^	0.009*
Overbite	3.39 (1.10)^A^	3.13 (0.34)^A^	3.63 (0.50)^B^	0.002*
3-3 width	24.67 (2.01)^A^	23.12 (0.88)^B^	23.23 (1.16)^B^	0.006*
4-4 width	32.35 (2.41)	33.31 (2.25)	32.93 (2.01)	0.449
5-5 width	36.78 (2.66)	37.82 (3.20)	37.08 (2.88)	0.377
6-6 width	42.88 (3.00)	43.76 (3.24)	42.82 (3.11)	0.232

* Statistically significant at *p*< 0.05. Different letters in a row indicate the presence of a statistically significant difference between the groups.

Comprehensive orthodontic treatment was carried out with preadjusted 0.022 x 0.028-in Roth prescription appliance and mandibular incisor extraction. The archwire sequence was as follows: 0.014-in and 0.016-in NiTi round archwires, 0.017 x 0.025-in and 0.019 x 0.025-in NiTi, and 0.019 x 0.025-in stainless steel archwires. As part of the treatment, leveling and alignment were performed, as well as correction of the curve of Spee, closure of the extraction space, intercuspation and finishing. The tooth-size discrepancy created by the incisor extraction, when confirmed through Bolton analysis, was compensated with maxillary incisor enamel reduction. No dental stripping was performed in the mandibular dentition. 

**Figure 1:**
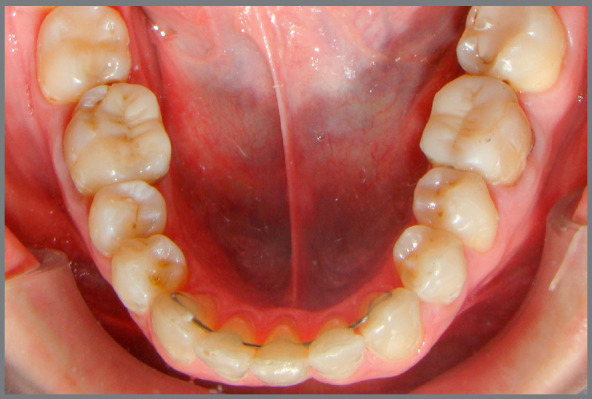
Fixed retainer.

At the end of treatment, all patients used a maxillary removable retainer (Hawley plate) and a 3x3 fixed retainer made with thick (0.025-in) round stainless steel wire bonded in all teeth from right to left mandibular canine (3-3) (Fig 1). All patients presented adequate protrusive anterior guidance and disocclusion lateral guidance in group function at the end of treatment.

At 1 year posttreatment follow-up, all patients still had the 3x3 bonded mandibular retainer in place. This information was obtained from the patients’ records. In the last follow-up recall (T_3_), some patients still had the 3x3 bonded mandibular retainer in place and some, due to personal reasons, did not. Thus, the sample was divided into two groups, according to the presence of the mandibular retainer, allowing the comparison of mandibular anterior crowding relapse between the two groups, as follows:

» Group 1: 9 patients who did not presented the 3x3 bonded mandibular retainer in place at T_3_. The mean (±SD) pretreatment, posttreatment and long-term follow-up ages were 21.96 ± 8.34, 23.99 ± 8.14 and 28.67 ± 8.04 years, respectively. The mean treatment time was 2.02 ± 0.5 years, and the mean long-term follow-up was 4.68 ± 1.41 years. The patients were without the fixed retainer for at least two years posttreatment.
» Group 2: 7 patients who presented 3x3 bonded retainer in place at T_3_. The mean (±SD) pretreatment, posttreatment and long-term follow-up ages were 25.37 ± 10.41, 27.45 ± 10.19 and 31.96 ± 9.56 years, respectively. The mean treatment time was 2.08 ± 0.42 years, and the mean long-term follow-up was 4.50 ± 2.42 years.

## METHODS

Pretreatment (T_1_), posttreatment (T_2_) and long-term follow-up (T_3_) dental casts were evaluated. All dental cast measurements were performed with a 0.01 mm precision digital caliper (Model/code 500-143B, Mitutoyo Sul America, São Paulo/SP, Brazil) by a single calibrated examiner. The following measurements were performed in the three stages (T_1_, T_2_ and T_3_).

**Figure 2:**
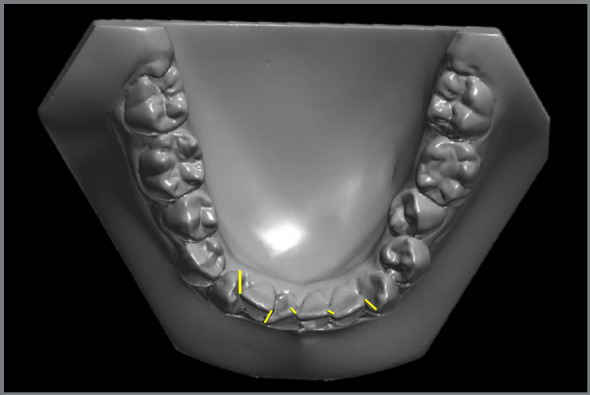
Little Irregularity Index: The sum of linear displacement of the anatomic contact points of the six anterior teeth.

**Figure 3:**
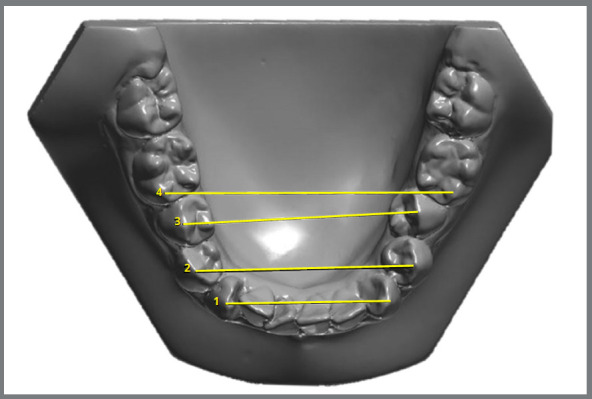
Interdental distances.

» Little Irregularity index, as described by Little^26^ (Fig 2)
» Overjet: linear distance between the most anterior point of the maxillary central incisor and the corresponding reference point on the mandibular incisor.
» Overbite: measured between the edge of the uppermost vertically erupted central incisor and the corresponding incisal edge of the opposite mandibular tooth, perpendicular to the occlusal plane.
» 3-3 width: distance between the crown tips of the right and left mandibular canines (Fig 3).
» 4-4 width: distance between the cusp tips of the mandibular first premolars (Fig 3).
» 5-5 width: distance between the cusp tips of the mandibular second premolars (Fig 3).
» 6-6 width: distance between the mesiobuccal cusp tips of the mandibular first molars (Fig 3).

### ERROR STUDY

One month after the first measurement, 64 dental casts were randomly selected and remeasured by the same examiner. The random errors were calculated according to Dahlberg’s formula,^27^ and the systematic errors were evaluated with dependent t-tests.^28^

### STATISTICAL ANALYSIS

Normal distribution was verified with Kolmogorov-Smirnov tests. Since all variables showed normal distribution, parametric tests were used.

Repeated measures ANOVA, followed by a Tukey test when necessary, were used for the comparison of the variables at T_1_, T_2_ and T_3_.

Intergroup comparison of the Little index and arch shape variables in all stages and phases evaluated was performed with independent *t*-tests.

As the sample size had a reduced number and was subdivided into two groups, a power test was calculated for independent *t*-test, giving a power of 0.80. 

All statistical analyses were performed with Statistica software (Statistica for Windows 7.0; Statsoft, Tulsa, Okla, USA). Results were considered statistically significant at *p*< 0.05.

## RESULTS

The random errors varied from 0.15 (Little Irregularity Index) to 0.31 (3-3 width). There was no significant systematic error. 

Little Irregularity Index was significantly reduced with treatment, and showed a significant relapse at follow-up (Table 1). Overjet was corrected with treatment and remained stable in the follow-up. Overbite was maintained with treatment and increased significantly in the follow-up. Intercanine width was significantly reduced at T_2_ and remained stable at T_3_ (Table 1). The 4-4, 5-5 and 6-6 widths presented similar patterns of change, increasing with treatment and showing a slight decrease at the long-term follow-up (Table 1).

Ages, treatment time and long-term follow-up evaluation were comparable in both groups (Table 2).

**Table 2:** Results of the intergroup comparability of initial, final and long-term evaluation ages, treatment time and long-term follow-up evaluation (t-tests).

Variables (years)	Group 1 No bonded 3x3 retainer (n = 9)		Group 2 3x3 bonded retainer (n = 7)		*p*
	Mean	SD	Mean	SD	
Pretreatment age (T_1_)	21.96	8.34	25.37	10.41	0.474
Posttreatment age (T_2_)	23.99	8.14	27.45	10.19	0.462
Follow-up age (T_3_)	28.67	8.04	31.96	9.56	0.467
Treatment time (T_2_-T_1_)	2.02	0.50	2.08	0.42	0.805
Time of posttreatment evaluation	4.68	1.41	4.50	2.42	0.859

The groups were comparable regarding Little Irregularity Index at pretreatment (Table 3). Both groups showed similar crowding correction at posttreatment (Table 3). Mandibular Little Irregularity Index was significantly greater in the group without retainer at postretention period, when compared to the retainer group (Table 3). The groups were comparable regarding overjet at all stages (Table 3). 

**Table 3:** Results of the intergroup comparison of the Little Irregularity Index, overjet and overbite in the stages and periods evaluated (t-tests).

Variables (mm)	Group 1 No bonded 3x3 retainer (n = 9)		Group 2 3x3 bonded retainer (n = 7)		*p*
	Mean	SD	Mean	SD	
Initial Little Irregularity Index (T_1_)	8.53	1.17	9.36	1.37	0.211
Final Little Irregularity Index (T_2_)	0.29	0.14	0.18	0.07	0.095
Follow-up Little Irregularity Index (T_3_)	2.27	0.80	0.88	0.73	0.003*
Little Irregularity Index treatment change (T_2_-T_1_)	-8.24	1.23	-9.18	1.42	0.179
Little Irregularity Index follow-up change (T_3_-T_2_)	1.98	0.92	0.70	0.72	0.009*
Overjet (T_1_)	3.99	0.62	3.91	1.30	0.859
Overjet (T_2_)	3.13	0.45	2.75	0.41	0.106
Overjet (T_3_)	3.34	0.81	2.97	0.28	0.273
Overjet treatment change (T_2_-T_1_)	-0.87	0.84	-1.16	1.63	0.650
Overjet follow-up change (T_3_-T_2_)	0.21	0.60	0.23	0.35	0.965
Overbite (T_1_)	3.55	0.74	3.18	1.48	0.524
Overbite (T_2_)	3.04	0.29	3.24	0.39	0.268
Overbite (T_3_)	3.85	0.49	3.34	0.37	0.040*
Overbite treatment change (T_2_-T_1_)	-0.51	0.68	0.06	1.64	0.362
Overbite follow-up change (T_3_-T_2_)	0.80	0.33	0.10	0.09	0.000*

*Statistically significant at *p*< 0.05.

Overbite was significantly greater in the group without retainer at T_3_ (Table 3). The changes in overbite from posttreatment to long-term follow-up were greater in the group without retainer (Table 3). 

There was no statistically significant difference for the mandibular 3-3; 4-4; 5-5 and 6-6 widths in all times and periods evaluated between retainer and no retainer groups (Table 4).

**Table 4:** Results of the intergroup mandibular arch transversal distances comparison in the stages and periods evaluated (t tests).

Variables (mm)	Group 1 No bonded 3x3 retainer (n = 9)		Group 2 3x3 bonded retainer (n = 7)		*p*
	Mean	SD	Mean	SD	
3-3 width (T_1_)	24.08	1.06	25.44	2.73	0.192
3-3 width (T_2_)	23.17	0.73	23.06	1.11	0.816
3-3 width (T_3_)	23.39	0.80	23.04	1.56	0.564
3-3 width treatment change (T_2_-T_1_)	-0.91	0.78	-2.37	2.03	0.066
3-3 width follow-up change (T_3_-T_2_)	0.22	0.23	-0.02	1.03	0.497
4-4 width (T_1_)	32.73	1.64	31.87	3.23	0.495
4-4 width (T_2_)	32.89	1.43	33.86	3.04	0.411
4-4 width (T_3_)	32.72	1.71	33.20	2.47	0.650
4-4 width treatment change (T_2_-T_1_)	0.15	1.67	1.99	4.42	0.268
4-4 width follow-up change (T_3_-T_2_)	-0.16	0.68	-0.65	2.59	0.595
5-5 width (T_1_)	37.00	2.96	36.50	2.40	0.726
5-5 width (T_2_)	37.16	3.27	38.68	3.12	0.363
5-5 width (T_3_)	36.86	3.00	37.36	2.92	0.747
5-5 width treatment change (T_2_-T_1_)	0.15	2.63	2.17	3.22	0.189
5-5 width follow-up change (T_3_-T_2_)	-0.29	0.95	-1.32	3.18	0.369
6-6 width (T_1_)	43.17	3.42	42.50	2.56	0.674
6-6 width (T_2_)	43.61	3.57	43.96	3.02	0.838
6-6 width (T_3_)	43.35	3.21	42.13	3.07	0.455
6-6 width treatment change (T_2_-T_1_)	0.43	1.34	1.45	3.30	0.411
6-6 width follow-up change (T_3_-T_2_)	-0.25	0.48	-1.82	2.87	0.126

## DISCUSSION

The present work is the first study evaluating whether fixed retainer has any influence on relapse of anterior crowding in cases treated with mandibular incisor extraction. Færøvig and Zachrisson^29^ evaluated mandibular incisor extraction cases followed-up over 4.3 years; however, their study comprised Class III malocclusion with open bite tendencies, and no comparison was made between patients with and without retainers at the long-term posttreatment. 

The present sample was consisted of 16 subjects; yet, considering the difficulties of obtaining a homogeneous sample from orthodontic treatment performed with mandibular incisor extraction,^17^ the number seems to be a reasonable amount. The follow-up stage agrees with the current studies, considering that about half of total relapse takes place in the first two years after debonding.^6^ ^,^ ^19^ Furthermore, it should be emphasized that, at 1-year posttreatment follow-up, all the patients still had 3x3 fixed retainer in place. So, the relapse that was observed in the group without retainer occurred after the first year postretention throughout the long-term follow-up.

The decision to extract one mandibular incisor in this study was based on the amount of mandibular anterior crowding that patients presented at the beginning of treatment, presence of Class I malocclusion and straight facial profile. The extraction of premolars was not considered an ideal treatment plan. These combination of factors favors an efficient and adequate orthodontic treatment plan, and is in accordance with the current literature.^12^ ^-^ ^14^

The initial Little Irregularity Index (8.89 ± 1.29) was significantly reduced with treatment (0.25 ± 0.12), and showed a significant increase (1.67 ± 1.03) in the follow-up (Table 1). This result is in agreement to Riedel et al,^25^ although their initial irregularity was less severe. Canut^16^ reported a greater relapse at follow-up, however, he evaluated only patients who were out of retention for at least 5 years. It must be taken into account that the present results were obtained from the whole sample (retainer/no retainer).

Overjet was corrected with treatment and remained stable in the follow-up (Table 1). Since patients had a Class I molar relationship, they had no sagittal discrepancy at pretreatment, and the decrease in overjet as a result of treatment was accounted at the expense of the alignment of the mandibular incisors and then remained stable at long-term follow-up. On the other hand, overbite was maintained with treatment, and increased significantly in the follow-up (Table 1). This result is in accordance to Bahreman^12^, where the extraction of mandibular incisors in the presence of overbite at the pretreatment is not indicated, because cases treated with this type of tooth extraction tend to increase the overbite at long-term.

The 3-3 width was significantly reduced with treatment and remained stable until the follow-up stage (Table 1). This result is different from the majority found in the literature, in which it was found that the 3-3 width reduced with treatment and continued to decrease postretention.^25^ ^,^ ^30^ It may be speculated that this reduction is due a tipping or body movement to a narrower part of the arch. Moreover, this contradicts what the studies say about one of the major advantages of incisor extraction, which is the maintenance of interdental distances, mainly the intercanine width.^12^

The subgroups were comparable regarding initial, final and follow-up ages, treatment time and follow-up stage (Table 2). The initial mean age was over 21 years old, and at the follow-up stage, patients were over 28 years. This finding shows that patients had no residual growth that may have influenced the relapse.^31^ ^,^ ^32^ The majority of long-term studies presents a sample with a lower initial age, and, sometimes the follow-up stage coincides with the end of growth, and crowding often cannot be differentiated from the occlusal maturational changes.^33^ ^,^ ^34^

In the follow-up stage, the subgroup without retainer presented a significant relapse when compared to the retainer subgroup (Table 3). According to Little, to obtain a significant value of crowding relapse in the postretention stage, the index must be greater than 3.5 mm. In the present study, the group without retention presented a mean (±SD) Little index of 2.27±0.80 at T_3_, and this is not considered a great crowding at the follow-up. However, this parameter was significantly greater than in Group 2. This result is in agreement with other studies^18^ ^,^ ^20^, in which mandibular anterior alignment was significantly better for the group using a 3x3 fixed retainer. This result was already expected, since the 3x3 bonded retainer aims at keeping the alignment and preventing relapse. However, Lang et al^19^ found that some degree of relapse could be observed even in patients with long-term bonded retention.^35^ ^,^ ^36^ According to Little et al,^9^ the only way to ensure continued satisfactory alignment posttreatment probably is by the use of fixed retention for lifetime.

The group without retainer presented greater overbite at T_3_ than the retainer group, and the changes in overbite from posttreatment to the long-term follow-up stage were greater in the no-retainer group (Table 3). This increase in overbite is expected in mandibular extraction cases.^12^ However, the no retainer group presented greater overbite and significant relapse of mandibular anterior crowding, when compared to the retainer group. According to Francischoni et al.^37^ there is a positive correlation of the relapse of mandibular incisor crowding with the increase of overbite in the long-term. It could be thus speculated that the presence of fixed retainer decreased the tendency for the increase in overbite at the long-term.

The interdental distances showed no difference at the follow-up stage between the no retainer and retainer subgroups (Table 4). This is in agreement with other studies.^36^ ^,^ ^38^ However, these studies did not evaluate orthodontic treatment performed with mandibular incisor extraction. There is no report in the literature comparing these measures in cases of incisor extraction with or without 3x3 bonded retainer at the follow-up stages.

### CLINICAL IMPLICATIONS

Despite the study suggesting that alignment stability seems to be better in incisor extraction cases than that achieved in cases subjected to premolar extraction,^16^ it was possible to observe a significant anterior crowding relapse in this study. Little^26^ stated that the evidence of progressive instability of the orthodontic treatment is always first noticed by the mandibular anterior crowding after the removal of the retainers. Since the alignment condition of the mandibular incisors appears to be a limiting factor in treatment and stability, it is recommended the use of fixed retainer in mandibular anterior teeth for lifetime.

## CONCLUSIONS

There was a significant relapse in cases treated with mandibular incisor extraction, at follow-up.

Patients without retainer showed a significant relapse in the follow-up, when compared to the retainer group.
